# Supplementary material for: Human Mutant Dynactin Subunit 1 Causes Profound Motor Neuron Disease Consistent with Possible Mechanisms Involving Axonopathy, Mitochondriopathy, Protein Nitration, and T-Cell-Mediated Cytolysis
Source: Biomolecules. 2025 Nov 21;15(12):1637. doi: 10.3390/biom15121637 (PMC12730482; doi:10.3390/biom15121637)
Supplement: Supplementary file 1 [file biomolecules-15-01637-s001.zip › biomolecules-3933869-supplementary.pdf]

Supplementary Figures

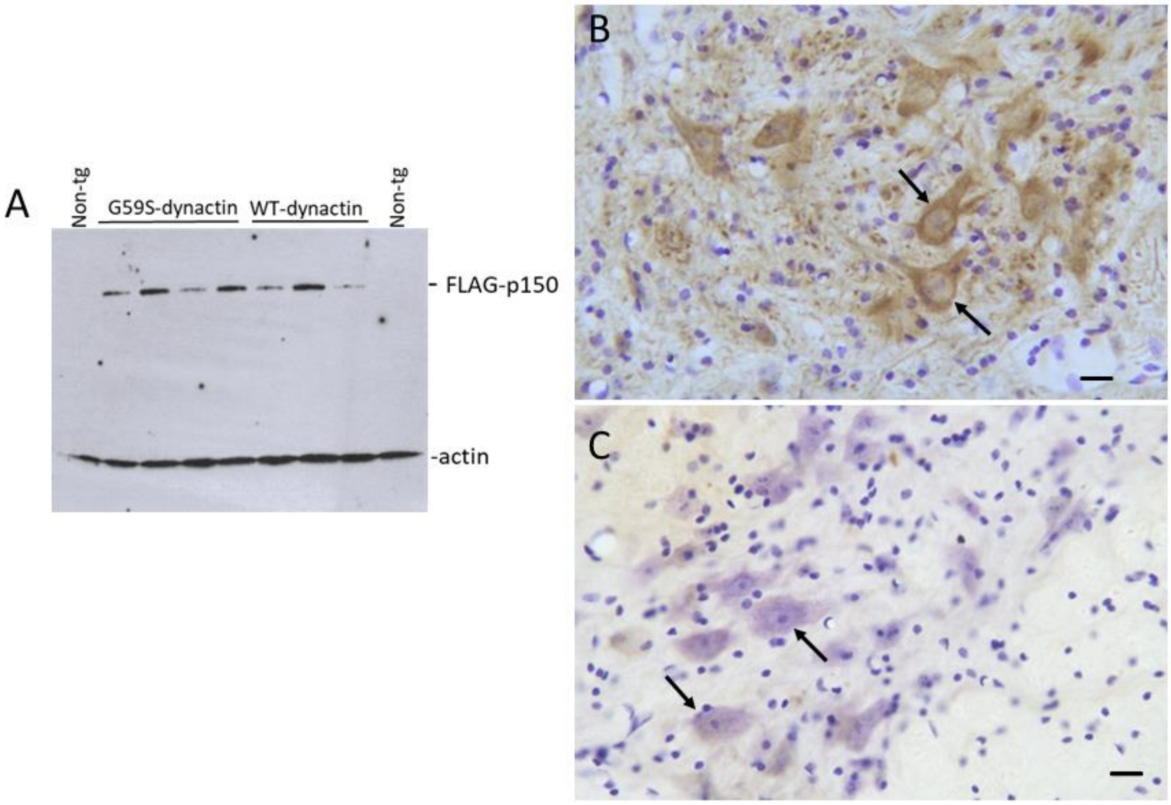

Figure S1. Validation of human DCTN1 rg mice. (**A**) Full-length western blot showing FLAG tagged human DCTN1 in G59S-DCTN1 and wildtype-DCTN1 tg mice. The mobility of the FLAG immunoreactive protein is consistent with p150-dynactin. Immunoreactivity was not detected in non-tg mouse lanes. Western blot reprobe showing actin immunoreactivity in all lanes. (**B**,**C**). Immunohistochemistry shows that FLAG immunoreactivity is detected in spinal cord motor neurons in tg mice (**B**) but not in non-tg mice (**C**).

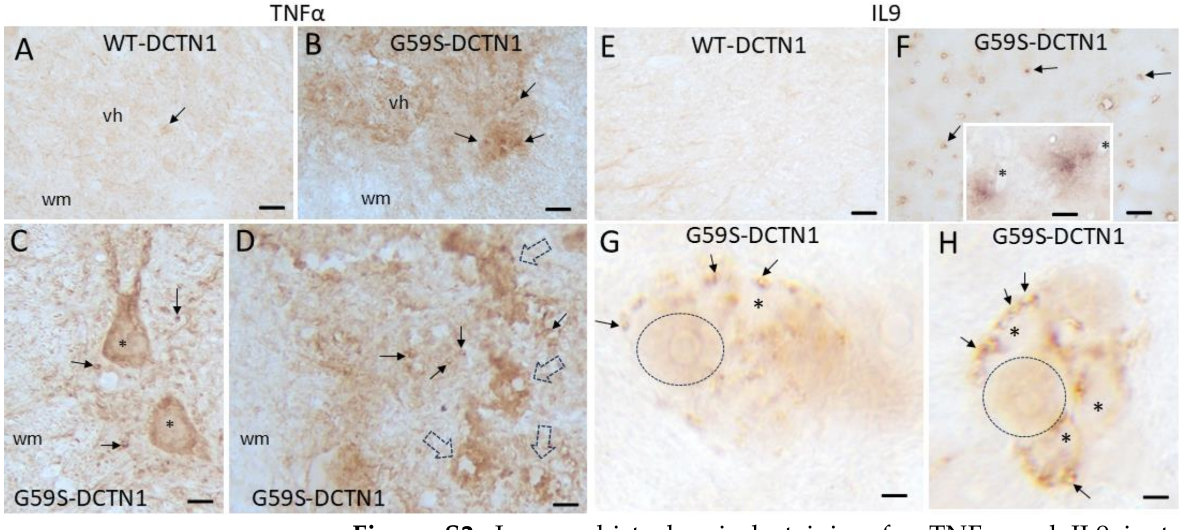

Figure S2: Immunohistochemical staining for TNFα and IL9 in tg mouse spinal cord. (A) WT-DCTN1 sections had very low levels of

TNFα immunoreactivity in ventral horn (vh) and nearby white matter (wm). Occasionally faintly labeled cells could be discerned (arrow). (B) G59S-DCTN1 mouse section showed enrichment of TNFα immunoreactivity is spinal cord ventral horn (vh) neuropil and adjacent white matter (wm). In mice at early-stage disease some small TNFα-positive cells were seen as isolated cells or small clusters (nests) of cells (arrows). (C) Motor neurons with nascent feature of degeneration, such as small vacuolation, in G59S-DCTN1 mice became TNFα-positive (*) and small cells in the neuropil were also TNFα-positive (arrows). (D) In advanced stages of disease, the TNFα-positive motor neurons had large vacuoles and appeared swollen and lytic (open arrows). Nearby small cells in the ventral horn (vh) neuropil were also TNFα-positive (arrows). (E) IL9 immunoreactivity in WT-DCTN1 mouse spinal cord sections was very low. (F) In mice at early-stage disease perivascular IL9-positive cells accumulated as isolated cells or cell clusters (arrows). Inset show perivascular IL9-positive cell clusters. Capillaries are identified (*). (G,H) Structural damage to motor neurons was associated with the accumulation of small perineuronal IL9-postive cells decorating the surface of vacuolated (*) neurons (arrows). As the cytoplasmic vacuolation (*) became more severe, the perineuronal encrustation of small IL9-positive cell became more severe (I, arrows). Scale bars: 30 μm (A,B,E,F), 12 μm (F inset), 10.5 μm (C,D), 22 μm (G,H).

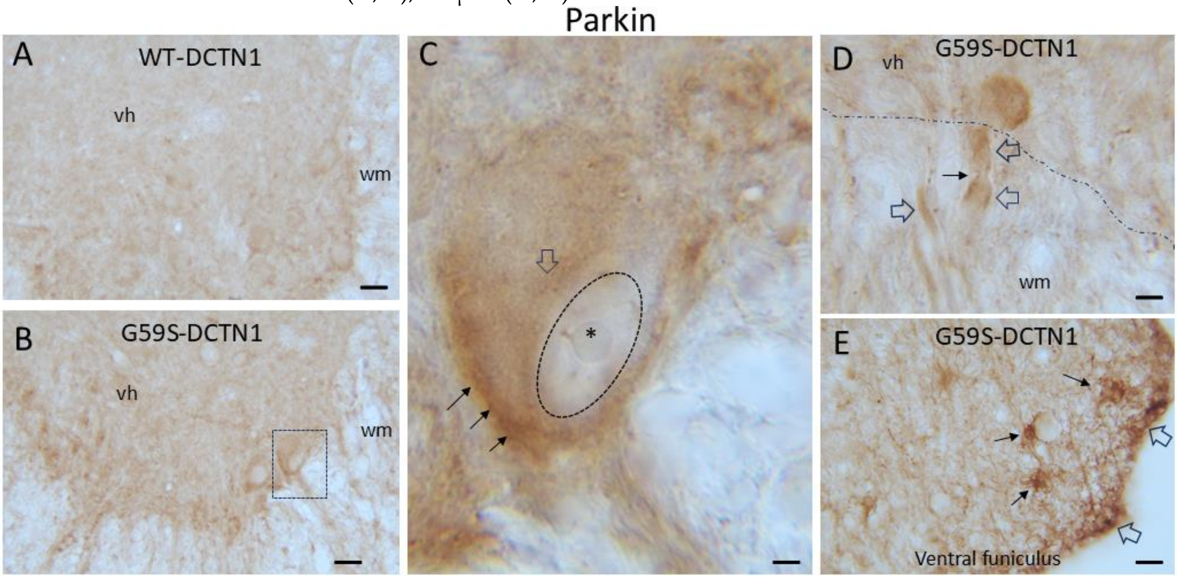

Figure S3: Immunohistochemical staining for Parkin in tg mouse spinal cord. (A,B) Parkin localizations in WT- and G59S-DCTN1 tg mouse motor neurons were generally similar in many neurons. Some modest accumulation of Parkin was seen specifically in chromatolytic motor neurons (B, dashed box shown in panel C). (C) In chromatolytic motor neurons as suggested by the rounded contour of the cell body and the eccentrically placed nucleus (dashed circle, * identified the nucleolus), Parkin immunoreactivity is marginated to the cell periphery (arrows) and diffuse particles are localized centrally in the cytoplasm (open arrow). (D,E) Prominent Parkin immunoreactivity was associated with degenerating/dystrophic axons near the transition from gray matter to white matter (dashed line) approaching the ventral root exit zone (D,

open arrows) and in reactive astrocytes (E, arrows). Scale bars: 30 μm (A,B), 4 μm, 8 μm (D), 12 μm (E).

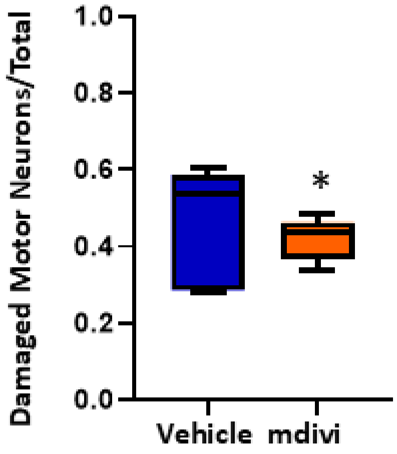

Figure S4: Box-whisker plot showing the ratio of damaged motor neuron to total motor neurons in G59S-DCTN1 mice treated with mdivi-1 or its vehicle (*, p=0.03).

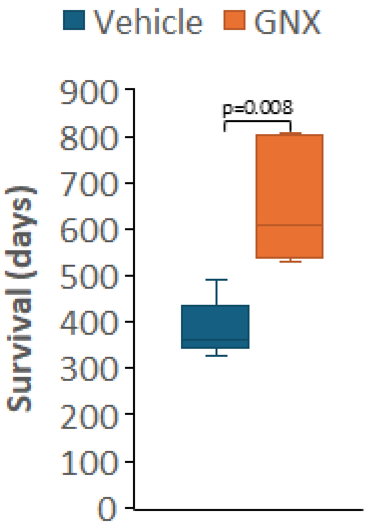

Figure S5: Box-whisker plot showing the survival of G59S-DCTN1 mice treated with GNX-4728 or its vehicle.
